# Pause at Your Own Peril: A Case Series on Rebound Pulmonary Hypertension

**DOI:** 10.7759/cureus.25552

**Published:** 2022-05-31

**Authors:** Sindhubarathi Murali, Subrat Khanal, Sanchari Banerjee, Omari Christie, Kartik Ramakrishna

**Affiliations:** 1 Department of Internal Medicine, State University of New York Upstate Medical University Hospital, Syracuse, USA; 2 Department of Pulmonary and Critical Care, State University of New York Upstate Medical University Hospital, Syracuse, USA; 3 Department of Diagnostic Radiology, State University of New York Upstate Medical University Hospital, Syracuse, USA

**Keywords:** restarting therapy, severe pulmonary arterial hypertension, interruption of therapy, rebound pulmonary arterial hypertension, pulmonary artery hypertension

## Abstract

Severe pulmonary arterial hypertension (PAH) is associated with high morbidity and mortality. Therapeutic approaches for intermediate- and high-risk pulmonary arterial hypertension have now shifted toward initial combination management, often including parenteral epoprostenol and iloprost and early assessment for a lung transplant. After the initiation of therapy, usually various combinations of different classes of medication, it is important to consider the potential interruption in therapy causing rebound PAH. We present two patients recently admitted to our hospital with rebound symptoms after interruption of their pulmonary vasodilator therapy.

## Introduction

Pulmonary arterial hypertension (PAH) encompasses a group of disorders resulting in elevated pulmonary arterial pressure (PAP), leading to right ventricular hypertrophy and failure [1]. Despite being such a detrimental disease, pharmaceuticals have improved patient symptoms, quality of life, and survival. The abrupt disruption of these medications can result in rebound pulmonary hypertension, right heart failure, and cardiogenic shock [2-4]. Although PAH literature has increased over the years, there is little data about rebound PAH after the brief discontinuation of medications and how to restart them. We present two cases where PAH medications were abruptly discontinued, resulting in right-sided heart failure.

## Case presentation

Case 1

The first case is a 41-year-old female, who was diagnosed with heritable PAH. She was on therapy with ambrisentan (10 mg daily), tadalafil (40 mg daily), and inhaled treprostinil (nine inhalations, four times daily), along with furosemide (40 mg twice daily). She had presented to the hospital with nausea and vomiting and was diagnosed with acute cholecystitis. For the past 48 hours, she was unable to tolerate her PAH medications. This resulted in severe dyspnea and worsening lower extremity edema. She was admitted to the surgical service; however, pulmonary medicine was consulted given her complex PAH history.

**Figure 1:**
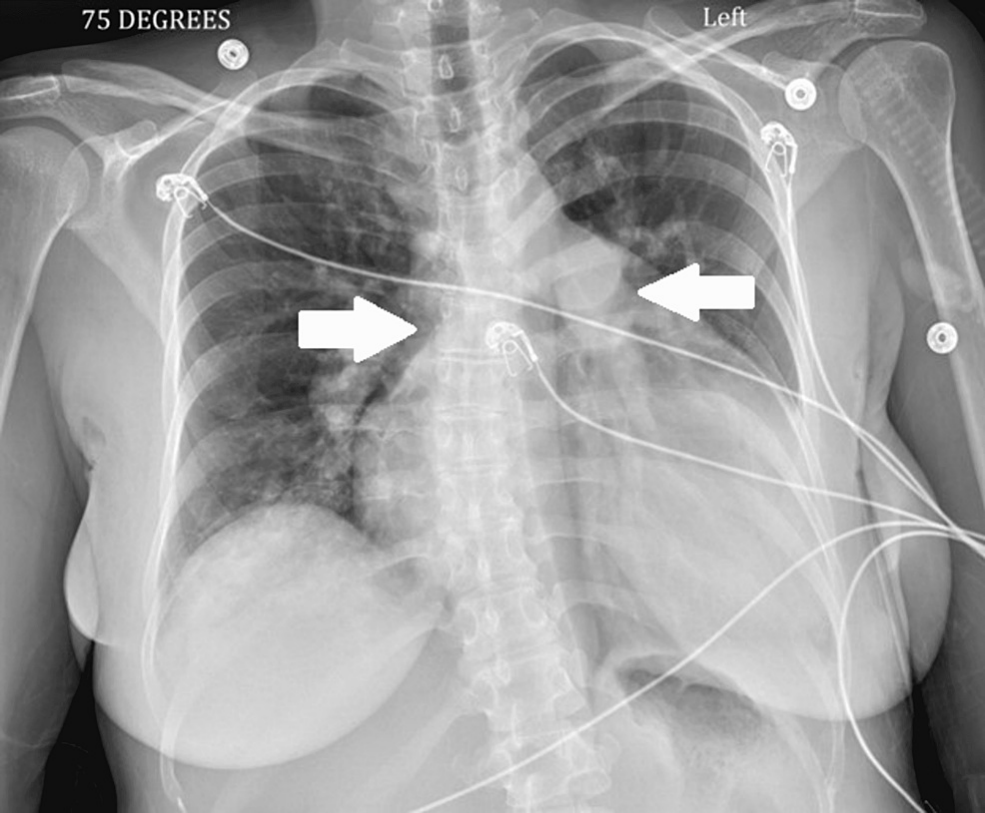
Case 1 chest X-ray demonstrating cardiomegaly and prominent pulmonary vasculature. Notice the ballooning of the right ventricle. Arrows indicate prominent pulmonary vasculature.

On physical examination, the patient was tachycardic and in moderate respiratory distress. She had a loud P2. An abdominal examination revealed tenderness in the right upper quadrant and voluntary guarding throughout the abdomen. Volume assessment revealed an elevated jugular venous pressure (JVP), presence of bilateral peripheral pitting lower extremity, and sacral edema. She was hypoxemic and required 3 L/minute of oxygen via nasal cannula. Chest radiograph showed cardiomegaly and prominent pulmonary vasculature, but no evidence of airspace disease (Figure 1).

**Figure 2:**
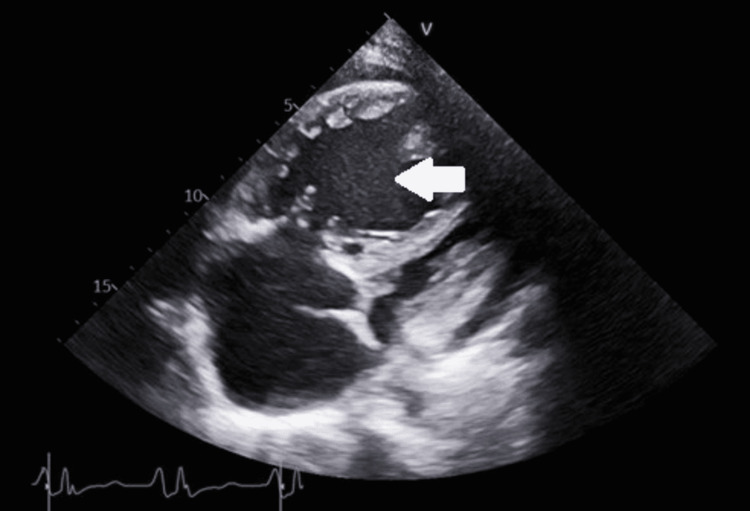
Case 1 transthoracic echocardiogram subcostal view. The arrow indicates dilated right ventricle comparable in size to the left ventricle.

Given the decompensated right heart failure, she was deemed to be at high risk for general anesthesia. The decision was made to treat the cholecystitis conservatively with antibiotics. She progressively required more oxygen within the first day of admission and was transferred to the medical ICU for a high-flow nasal cannula (50%/30 L). Transthoracic echocardiogram (TTE) showed a severely dilated right ventricle and right atrium (90.1 mL/m), severe tricuspid regurgitation (TR Vmax: 4.88 m/second; tricuspid annular plane systolic excursion (TAPSE): 1 cm), and an estimated right ventricular systolic pressure (RVSP) of 110 mmHg (Figure 2).

The left ventricle was small and under-filled with an ejection fraction of 70%. There was systolic and diastolic flattening of the interventricular septum.

**Figure 3:**
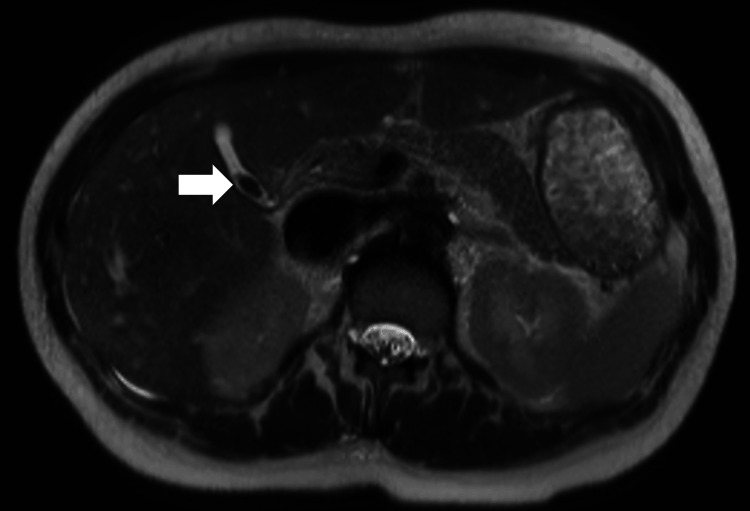
MRCP axial T2 haste showing an 11-mm filling defect in the gallbladder neck. The arrow indicates the defect in the gallbladder neck.

**Figure 4:**
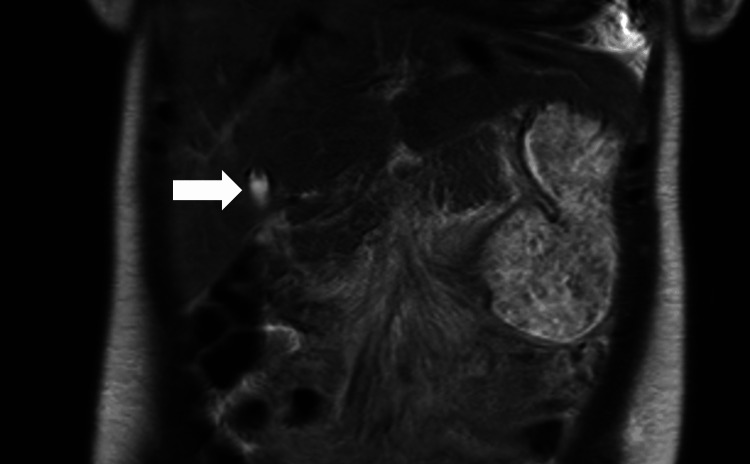
MRCP coronal T2 haste image showing the 11-mm stone in the gallbladder neck. The arrow indicates the stone in the gallbladder neck.

A furosemide infusion was started for aggressive diuresis. Her home medication of ambrisentan, tadalafil, and inhaled treprostinil was restarted at full doses. On day 8 of her hospital admission, she was net negative 9 L and could tolerate lying flat for the magnetic resonance cholangiopancreatography (MRCP). It revealed an 11-mm stone in her gallbladder neck without features of cholecystitis (Figure 3 and Figure 4).

There were no signs of choledocholithiasis or biliary ductal dilatation. An urgent surgical intervention was no longer required given her elevated risk of perioperative adverse events. She was able to tolerate all her oral medications and was discharged for outpatient follow-up with her pulmonologist and general surgeon for elective cholecystectomy. On discharge, the patient did not require any oxygen supplementation; however, a six-minute walking test (6MWT) performed at her outpatient pulmonologist’s office showed hypoxemia. She required 3 L of oxygen via nasal cannula with exertion.

Case 2

The second case is a 55-year-old female with neurofibromatosis type I who was first diagnosed with PAH in November 2020. The TTE at that time showed a tricuspid regurgitant jet of 4.6 m/second with an estimated RVSP of 100 mmHg, TAPSE of 1.6 cm, significantly dilated RV (4.8 cm) and RA (5.4 cm), a preserved LV ejection fraction of 62%, LA (1.7 cm), and flattening of the intraventricular septum. Right heart catheterization showed right atrial pressure (RAP) of 10 mmHg, RVSP of 86/10 mmHg, PAP of 87/30 (55) mmHg, pulmonary capillary wedge pressure (PCWP) of 5 mmHg, cardiac output/cardiac index (CO/CI) (Fick principle) of 2.54/1.67, and pulmonary vascular resistance (PVR) of 20 Wood units. She was categorized as severe PAH and started on an incremental dose of intravenous treprostinil, macitentan (10 mg), riociguat (0.5 mg), furosemide (20 mg), and spironolactone (25 mg). Riociguat was discontinued due to severe orthostatic hypotension. Her intravenous treprostinil was gradually increased to a goal of 65 ng/kg/minute.

**Figure 5:**
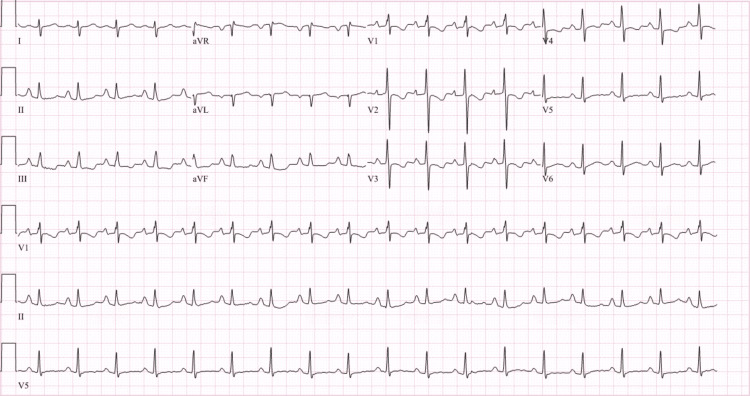
EKG showing nonspecific T wave inversions in aVL and V1 to V3.

Unfortunately, due to an empty cartridge, the patient had been without treprostinil infusion for 50 minutes. Over the next 12 hours, she developed dyspnea and chest tightness. She required 2 L/minute of oxygen via nasal cannula to maintain her oxygen saturation above 90%. On physical examination, she was visibly tachypneic and using accessory muscles. JVP was visibly elevated. P2 was prominent on the cardiac examination. EKG showed nonspecific T wave inversions in aVL and V1 to V3 (Figure 5).

**Figure 6:**
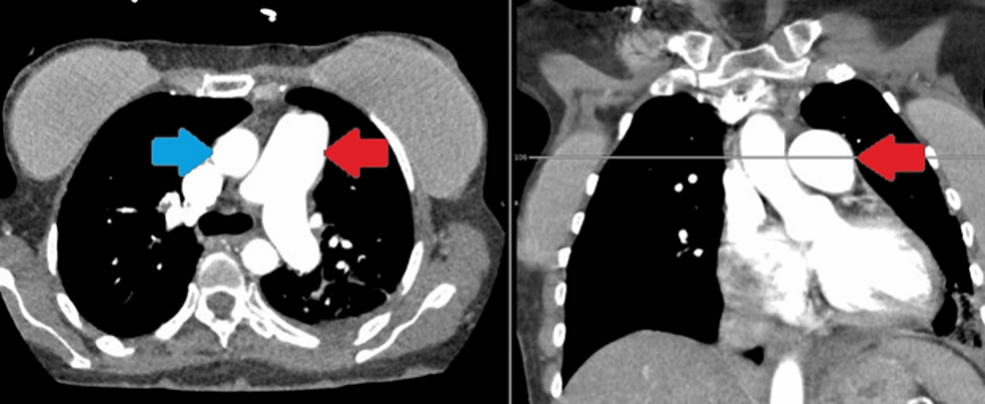
Contrast tomography (CT) of the thorax for case 2 demonstrating enlarged pulmonary arteries (red arrow) in comparison to the ascending aorta (blue arrow).

A contrast tomography (CT) of the thorax demonstrated enlarged pulmonary arteries without evidence of pulmonary embolism (Figure 6).

**Figure 7:**
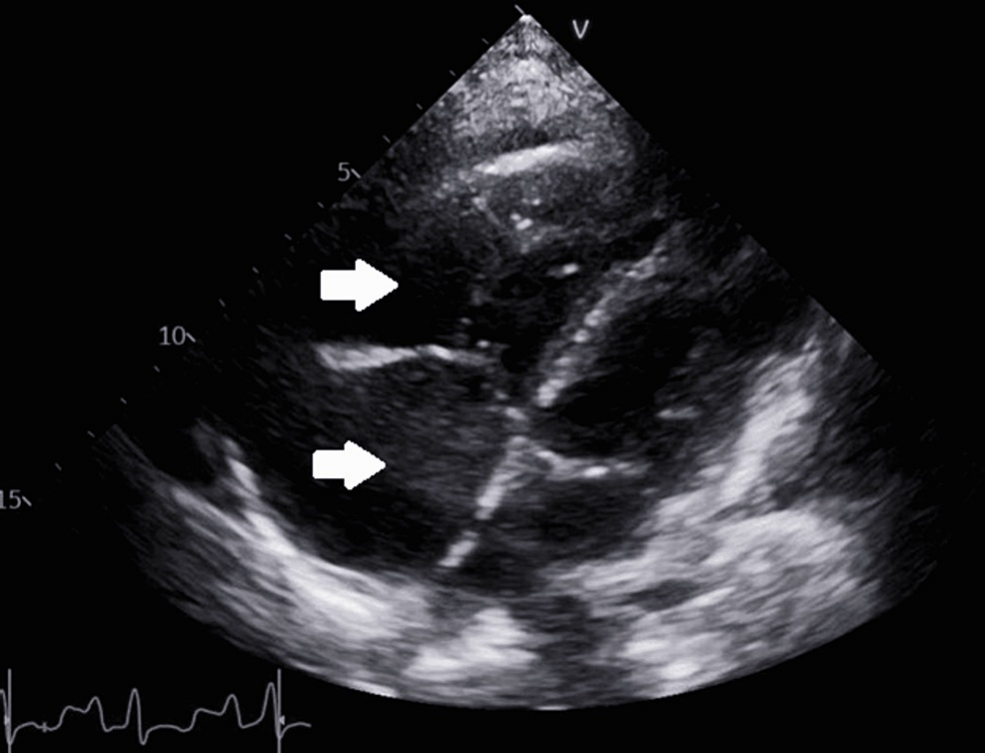
Four-chamber transthoracic echocardiogram for case 2. White arrows demonstrate an enlarged right atrium and ventricle in comparison to the left.

A TTE showed a markedly dilated RV with reduced systolic function (Figure 7).

The LV was found to be within normal limits. There was systolic and diastolic flattening of the interventricular septum, with moderate tricuspid regurgitation with a jet velocity of 4.3 m/second and TAPSE of 2.0 cm. Macitentan was restarted at a full dose of 10 mg daily, and IV treprostinil was resumed at her dose of 45 ng/kg/minute with continued biweekly up-titration in the hospital. Initially, she was diuresed with IV furosemide 40 mg twice daily.

Right heart catheterization showed RAP of 6 mmHg, RVP of 74/10 mmHg, PAP of 73/25 (47) mmHg, PCWP of 10 mmHg, CO/CI of 2.4/1.6 (Fick principle), and PVR of 15 Wood unit consistent with severe precapillary pulmonary hypertension. The decision was made to add a phosphodiesterase-5 (PDE5) inhibitor, tadalafil 20 mg once daily, with a plan to up-titrate to 40 mg daily. Her dyspnea and hypoxemia improved with eight days of diuresis, resumption of macitentan 10 mg PO daily, IV treprostinil (45 ng/kg/minute), and the addition of tadalafil 20 mg daily. She was discharged home on room air.

## Discussion

Pulmonary arterial hypertension (PAH) is a chronic, progressive, and life-threatening disease. PAH is a type of pulmonary hypertension that primarily affects the pulmonary arterioles. There are many different etiologies for PAH; however, the common pathogenesis of the disease is related to vascular remodeling, endothelin dysfunction, and smooth muscle proliferation, resulting in pulmonary artery vasoconstriction [5]. Severe PAH can lead to right heart failure, hemodynamic compromise, and eventually death.

In recent years, the understanding of the pathogenesis of PAH has led to the development of many therapeutic targets. The discontinuation of one class of medication may be more detrimental than another. Phosphodiesterase-5 (PDE5) inhibitors such as sildenafil act by preventing the deterioration of nitric oxide (NO) in the vascular smooth muscle, resulting in vasodilation [5]. The withdrawal of sildenafil results in rapid clinical deterioration, acute right heart failure, and a decrease in the six-minute walking test (6MWT) [6]. Endothelin receptor antagonists (ERAs) prevent endothelin-1 from exerting its effect on smooth muscle cells via the ET-A and ET-B receptors, causing vasoconstriction and cell proliferation. There is limited knowledge of the sudden cessation of ERAs [5]. Calcium channel blockers (CCBs) are used in a small percentage of patients who have positive vasoreactivity testing to calcium channel blockers (CCB) or pulmonary vasodilators such as nitric oxide during right heart catheterization [7]. Clinical deterioration can result from the discontinuation of CCBs, which is why it is recommended to start pulmonary vasodilatory therapy prior to stopping CCBs [8]. The final class of pharmaceuticals is the prostacyclin analogs, which act on prostaglandin I (IP) receptors, leading to the vasodilation of the pulmonary artery. These include the PGI2 analogs and PGI2 IP receptor agonist, selexipag [5]. The rapid discontinuation of this medication has catastrophic consequences, including rapid right heart failure and cardiogenic shock [2-4]. The potency of prostacyclin medications is the reason for the aggressive withdrawal symptoms.

After the abrupt withdrawal of medication, patients should be restarted on their home medication promptly. If discontinuation of the medication is less than 4-5 half-lives of the last dose taken, then the medication should be taken at the last tolerated dose. If the time between doses is greater than 4-5 half-lives, then a lower dose with rapid up-titration based on tolerance is required [5]. In the case of sildenafil (t½ = 16-20 hours) or tadalafil (t½ = 60-75 hours), we would restart sildenafil < 20 mg every eight hours and tadalafil < 40 mg/day if greater than 4-5 half-lives had elapsed between doses [5]. A similar situation would be the case for ERAs and CCB with the assessment of the risk versus the benefits of restarting at the full dose [5]. In the case of parenteral PGI2 analogs, when there has been a prolonged interruption, it is dangerous to re-titrate the drug from the lowest dose. It is recommended to start at a dose 10% lower and titrate as tolerated [5]. For inhaled treprostinil or oral treprostinil (t½ = four hours) or epoprostenol (t½ = 3-6 minutes), the risk versus the benefits of resuming the same or lower doses should be considered if greater than 4-5 half-lives have elapsed [5,9-11]. Selexipag should be started at a lower dose and titrated up if the interruption of the drug is greater than 4-5 half-lives (65 hours) [12].

In the two cases presented, the abrupt discontinuation of the patients’ treprostinil (PGI2 analog) resulted in their rapid decline. In the first case, the deterioration was further exacerbated by their inability to take their PO medications of ambrisentan and tadalafil. A predictor of their rapid decompensation was the severity of their underlying illness. The first patient was recently determined to be a WHO functional class III as she was unable to climb a single flight of stairs without multiple pauses. The second patient had an initial mean PAP of 55 mmHg, CI of 1.67, and PVR of 20 Wood units, suggestive of severe pre-capillary PAH. She was only on IV treprostinil for a short period of time before her pump had malfunctioned. Thus, her hemodynamics had likely not significantly changed from the time of her PAH diagnosis. At the time of hospitalization with rebound PAH, she was noted to have declining cardiac output. Both patients were restarted on their home regiments as soon as they could take their medications. Despite this, they went into right heart failure and required aggressive diuresis. The second patient’s regimen was further escalated by adding tadalafil.

## Conclusions

These cases highlight the severity of abrupt discontinuation of PAH medication and in turn provide some guidance on how to manage such patients in the inpatient setting. It is crucial to restart pulmonary vasodilator therapy as soon as possible. The dose to restart certain medication is dependent on the class of PAH therapy and the duration the medication has been withheld.
